# Estimating the effect of non-pharmaceutical interventions to mitigate COVID-19 spread in Saudi Arabia

**DOI:** 10.1186/s12916-022-02232-4

**Published:** 2022-02-07

**Authors:** Donal Bisanzio, Richard Reithinger, Ada Alqunaibet, Sami Almudarra, Reem F. Alsukait, Di Dong, Yi Zhang, Sameh El-Saharty, Christopher H. Herbst

**Affiliations:** 1 RTI International, Washington, D.C., USA; 2 Epidemiology and Public Health Division, School of Medicine, University of Nottingham, Nottingham, UK; 3 Saudi Public Health Authority, Riyadh, Saudi Arabia; 4 Ministry of Health, Riyadh, Saudi Arabia; 5 College of Applied Medical Sciences, King Saud University, Riyadh, Saudi Arabia; 6 World Bank, Washington, D.C., USA

**Keywords:** SARS-CoV-2, COVID-19, Non-pharmaceutical interventions, Individual-based model, Saudi Arabia

## Abstract

**Background:**

The Kingdom of Saudi Arabia (KSA) quickly controlled the spread of SARS-CoV-2 by implementing several non-pharmaceutical interventions (NPIs), including suspension of international and national travel, local curfews, closing public spaces (i.e., schools and universities, malls and shops), and limiting religious gatherings. The KSA also mandated all citizens to respect physical distancing and to wear face masks. However, after relaxing some restrictions during June 2020, the KSA is now planning a strategy that could allow resuming in-person education and international travel. The aim of our study was to evaluate the effect of NPIs on the spread of the COVID-19 and test strategies to open schools and resume international travel.

**Methods:**

We built a spatial-explicit individual-based model to represent the whole KSA population (IBM-KSA). The IBM-KSA was parameterized using country demographic, remote sensing, and epidemiological data. A social network was created to represent contact heterogeneity and interaction among age groups of the population. The IBM-KSA also simulated the movement of people across the country based on a gravity model. We used the IBM-KSA to evaluate the effect of different NPIs adopted by the KSA (physical distancing, mask-wearing, and contact tracing) and to forecast the impact of strategies to open schools and resume international travels.

**Results:**

The IBM-KSA results scenarios showed the high effectiveness of mask-wearing, physical distancing, and contact tracing in controlling the spread of the disease. Without NPIs, the KSA could have reported 4,824,065 (95% CI: 3,673,775–6,335,423) cases by June 2021. The IBM-KSA showed that mandatory mask-wearing and physical distancing saved 39,452 lives (95% CI: 26,641–44,494). In-person education without personal protection during teaching would have resulted in a high surge of COVID-19 cases. Compared to scenarios with no personal protection, enforcing mask-wearing and physical distancing in schools reduced cases, hospitalizations, and deaths by 25% and 50%, when adherence to these NPIs was set to 50% and 70%, respectively. The IBM-KSA also showed that a quarantine imposed on international travelers reduced the probability of outbreaks in the country.

**Conclusions:**

This study showed that the interventions adopted by the KSA were able to control the spread of SARS-CoV-2 in the absence of a vaccine. In-person education should be resumed only if NPIs could be applied in schools and universities. International travel can be resumed but with strict quarantine rules. The KSA needs to keep strict NPIs in place until a high fraction of the population is vaccinated in order to reduce hospitalizations and deaths.

**Supplementary Information:**

The online version contains supplementary material available at 10.1186/s12916-022-02232-4.

## Background

In December 2019, a surge in viral respiratory infections was reported in Wuhan, China [1]. A high proportion of cases needed intensive care due to respiratory complications, and many of those hospitalizations resulted in deaths [2]. The virus and disease were subsequently named severe acute respiratory syndrome coronavirus 2 (SARS-CoV-2) and coronavirus disease 2019 (COVID-19), respectively. Given the rapid spread of the virus globally [3], on March 12, 2020, the World Health Organization (WHO) declared COVID-19 a pandemic. The Middle East/North Africa (MENA) region was one of the first world regions hit by the global spread of the virus outside China. The first COVID-19 death in the MENA region was reported by Iran on February 19, 2020 [4], after which many other countries in the region started to report SARS-CoV-2 cases [5]. As of February 8, 2021, 5,140,158 cases had been reported in MENA, with more than half of them occurring in Iran, Iraq, Marocco, and the Kingdom of Saudi Arabia (KSA) [5] Rapid spread of cases in MENA was linked to travel to and from China and movement across MENA countries [4, 6].

To mitigate SARS-CoV-2 spread, minimize cases and deaths, as well as avoid health system collapse due to hospitalization of severe cases, countries started to devise and implement various non-pharmaceutical interventions (NPIs), given the absence of effective pharmaceutical treatment or a COVID-19 vaccine in the early phase of the pandemic [7]. NPIs adopted to reduce the circulation of SARS-CoV-2 included movement restrictions (at subnational, national, and international levels); national or sub-national lockdowns; closure of public spaces (e.g., schools, offices, shops, malls); banning of social gatherings; requiring wearing protective equipment, such as face masks and shields; hand hygiene; physical distancing; extensive population testing; quarantine or isolation; and contact tracing. Such NPIs have repeatedly been shown to be effective in controlling the spread of highly contagious infections such as influenza, Ebola, and SARS [8–11]. NPI adoption, their scale of implementation, population adherence, and level of enforcement have varied across countries, due to cultural and political differences. Mathematical models have shown that adopting NPIs could reduce the spread of SARS-CoV-2 by up to 70%, with reductions dependent on intervention type, level of government enforcement, and population adherence [12–14]. High variability in NPI approaches and their implementation has characterized the response to mitigate the spread of SARS-CoV-2 in the MENA region [15]. Countries such as Israel, Lebanon, Iran, and the United Arab Emirates implemented, with different timing, strict NPIs targeting social behavior (i.e., mask-wearing, physical distancing), full or partial lockdowns, and suspended international travel [15–17].

The KSA started to reduce international travel in February 2020, by suspending flight routes to and from China, as well as suspending international pilgrimage [18, 19]. On March 2, 2020, the first COVID-19 case in KSA was reported [20]. To reduce the probability of an outbreak and subsequent spread of SARS-CoV-2, KSA quickly adopted a number of NPIs following the report of this first COVID-19 case [18, 19]. KSA suspended international (March 15, 2020) and national travel (March 21, 2020), imposed local curfews (March 23, 2020), and closed public spaces (i.e., schools and universities [March 9, 2020], shops [March 23, 2020]), as well as limited religious gatherings (March 4, 2020). KSA also mandated all citizens to respect physical distancing and to wear face masks. KSA’s experience to respond to previous coronavirus outbreaks caused by Middle-East respiratory syndrome coronavirus (MERS-CoV) helped the country to rapidly implement NPIs against SARS-CoV-2 [21]. In June 2020, NPIs in KSA were eased, with commercial activities fully re-opened, sports activities resumed, religious and pilgrimage activities resumed with limited attendance, and national flights allowed [22]. Physical distancing and mask-wearing were still mandatory, with penalties for those violating the mandate. Schools and universities re-opened using online teaching during the first 7 weeks. The number of daily COVID-19 cases peaked during the second week of June 2020, with a maximum of 4919 cases, which was followed by a steady decline, reaching a plateau of ~ 400 cases per day during October and November 2020 [23]. This early and strict adoption of NPIs successfully reduced SARS-CoV-2 transmission following its introduction: as of November 11, 2020, KSA had reported 351,849 cases and 5590 deaths (case fatality rate [CFR] equal to 1.59%) [23]. Like other countries in which NPIs were able to control the spread of SARS-CoV-2, KSA has been looking for a strategy to move forward and ease NPIs by re-opening the country to international travel and having students attend school in person.

The spread of SARS-CoV-2 and the effect of NPIs have been studied using different modeling techniques such as statistical models, compartmental models, and individual-based models (IBM) [24–26]. Most of the IBM models used contact networks based on country demography to represent the interaction among people, and IBM models have already been used to investigate the impact of NPIs on the spread of COVID-19 in KSA [27]. However, these models assumed a ‘well-mixed’ population: they did not account for the high heterogeneity of people’s contacts nor the duration of these contacts—key factors in the transmission dynamics and spread of infectious diseases [28, 29].

We present the results of a spatio-temporal IBM representing KSA’s population to assess the effect of individual or combinations of NPIs on COVID-19, as estimated by the number of cases, hospitalizations, and deaths by the beginning of the summer of 2020. The model was also used to forecast the impact of school re-openings and the resumption of international travel. In addition, our study also simulated the effect of various NPI combinations, such as re-opening of schools with or without mask-wearing and contact tracing. Study results provide KSA policymakers with information that helps them plan future strategies to maintain the low circulation of SARS-CoV-2.

## Methods

### Model overview

The spread of SARS-CoV-2 in KSA was modeled using an IBM, representing the whole population of KSA (IBM-KSA), i.e., approximately 34 million people. The IBM-KSA’s structure is based on a contact network representing interactions among individuals in different settings such as households, schools, and workplaces. The use of a network structure instead of a pure agent model allows the IBM-KSA to account for contact heterogeneity and, at the same time, maintains a manageable level of abstraction, reducing computational load. The KSA-IBM simulates the heterogeneity of the number of contacts among people and the duration of these contacts. Available demographic data was used to build a simulated population similar to the actual KSA population, and the model accounts for the different contact interactions among various age groups [30–32]. NPIs adopted during the pandemic (as of November 20, 2020), as well as their timing were included in the KSA-IBM. Different NPI implementation approaches were tested to assess their impact on the spread of SARS-CoV-2. The simulated COVID-19 epidemics based on each scenario were used to estimate the effective reproduce number (*R*_*t*_) across a forecasted period. The estimated *R*_*t*_ was then used to calculate the forecasting of reported cases, hospitalizations, and deaths for each NPI scenario starting from the number of reported cases as of June 21, 2020. The modeled time period was from June 21, 2020, to June 21, 2021. We selected this time window to be able to capture the effect of a full year after the easing of NPIs. A detailed description of the IBM-KSA model structure and the model’s transmission parameters is provided in the Additional file 1 [33–51].

### KSA-IBM network structure

The KSA-IBM’s network structure is built to capture the heterogeneity of contacts among people, as seen in a real-world setting [28, 37]. Contact heterogeneity is one of the key drivers of infectious disease spread in communities. The network framework is built using a network with ‘scale-free’ and ‘small-world’ characteristics as described in several social networks [52]. A scale-free network is characterized by a high fraction of nodes (individuals) with few connections and a few nodes having a high number of connections. The nodes having high connectivity are often called ‘hubs’—in the field of infectious disease transmission dynamics the ‘hubs’ represent key individuals who can act as so-called super-spreaders. A network has a ‘small-world’ characteristic when two nodes in the network can reach each other through a short sequence of connected nodes (a so-called short path) [53]. Interactions among individuals occur in particular locations, which may have a key role in disease agents’ spread. The locations in which people spend most of their daily time are households, schools, and workplaces [39, 40]. Interactions outside these routine locations (e.g., markets, restaurants, and cinemas) are at the base of the ‘small-world’ characteristic of human social networks. The KSA-IBM assigns location attributes to each link to capture the different settings in which people have person-to-person encounters. The methods applied to create a ‘scale-free’ and ‘small world’ network for the KSA-IBM are fully described in Additional file 1.

The probability of a naïve individual to acquire SARS-CoV-2 infection from an infected and infectious individual is linked to the duration of their interaction. To include contact duration in the model, we added weights to node links, making the network a ‘weighted network’. In a weighted network, all links have a weight that describes the strength of the transmissible contact between two nodes [36]. In the KSA-IBM, the weight of a link is represented by the duration of the contact in minutes. A power-law distribution is used to describe the heterogeneity of contact duration among individuals, as used by social studies performed in different settings (Additional file 1) [36–38]. The distribution of contacts among individuals was estimated using a power law with *α* = 2.5 (Additional file 1) [35]. A time duration in minutes was assigned to each connection among two individuals. The duration of a connection was assigned generating the minutes from a power law distribution with *α* = 1.5 (Additional file 1) [37, 38]. Another critical factor that shapes the social network of an individual is age. People tend to have more interaction with individuals of the same age group, such as who they meet at school, in the workplace, or at recreational locations [41]. Thus, when building the KSA-IBM, we accounted for the interactions among people of different age-groups using contact matrices specific for KSA (Additional file 1) [41].

People’s travel between cities is a main driver of national and international SARS-CoV-2 spread [3]. The KSA-IBM accounts for movement flux across cities in KSA during the creation of the contact network. Because no data about people’s movement was available for KSA, we applied a general gravity model based on cities’ populations and distances. A similar model approach has been used to model the spread of Ebola during the outbreak that occurred in Western Africa during 2014–2016 (Additional file 1) [42]. The results of the gravity model were used to connect individuals to different locations [33, 43, 44].

### Epidemiological model

The epidemiological model implemented in the KSA-IBM uses the classical transition status seen in SEIR compartmental models: Susceptible (*S*) → Exposed (*E*) → Infectious (*I*) → Recovered/Dead (*R*). We calculated the hospitalization and fatality rates for KSA [23] to determine the number of infectious individuals who also would be at risk of being hospitalized or dying. The transition from one status to another is a function of pathogen characteristics (e.g., virulence, incubation period, infectious period, hospitalization, and fatality rate [Additional file 1]) and interaction among individuals (only for *S* to *I*) [46–49, 54]. The fraction of symptomatic cases is set equal to 20% [47].

### Simulated scenarios

The KSA-IBM was used to investigate the effect of NPIs on the spread of SARS-CoV-2 in KSA. Different scenarios were created to test the efficacy of NPIs adopted by KSA, as of November 11, 2020. Mask-wearing, physical distancing, and contact tracing were the NPIs tested using the KSA-IBM. We modeled the effect of these NPIs for the period after June 21, 2020, i.e., when the KSA lockdown NPI was lifted. All scenarios included a reduction of people interaction outside their household members equal to 15% [34] to represent changes in movement behavior after the lockdown had been lifted. The model was run to evaluate the impact of different scenarios on COVID-19 symptomatic cases, hospitalizations, and deaths. Described below are 15 hypothetical scenarios classified under 5 general NPI strategy groups tested with the KSA-IBM:

A. *No NPIs adopted by the country after June 21, 2020*. This scenario is the baseline model used to compare the effectiveness of NPIs. To account for voluntary self-protection behavior even in case of no NPIs being mandated by the government, we assumed that mask-wearing, physical distancing, and self-isolation were performed by 20% of symptomatic cases. The ‘baseline’ model also included school closures and international travel bans, which are NPIs that still were in place in KSA as of November 11, 2020.
B. *Mandatory mask-wearing and physical distancing adopted after June 21, 2020*. These NPIs reduce the infectious probability of giving protection outside the household. The probability of infecting other individuals was reduced by 40% when an infectious individual was wearing a mask [25]. Physical distancing reduced the transmission between individuals by 70% [55]. These scenarios represent the NPIs already in use in KSA. We tested four scenarios, because we do not have data on population adherence to mandatory mask-wearing and physical distancing, or of the effectiveness of the contact tracing. A recent study showed that approximately 50% of people follow physical distancing guidance [56] and more than 80% wear masks outside their home [57]. Thus, we decided to test a middle (50% of the population) and high (≥70%) adherence to physical protection guidance. KSA is adopting a digital platform to perform contact tracing. Four sub-scenarios were simulated with different population adherence to the guidance:
  1. Baseline | Mask-wearing: 80% | Physical distancing: 70%.
  2. Baseline | Mask-wearing: 50% | Physical distancing: 70%.
  3. Baseline | Mask-wearing: 80% | Physical distancing: 50%.
  4. Baseline | Mask-wearing: 50% | Physical distancing: 50%.
  The scenario does not include contact tracing; however, it does include self-isolation, which was assumed to be 20% of symptomatic cases. Self-isolation was set lower due to the mean size of KSA’s households, making effective self-isolation difficult.
C. *Contact tracing of infected people and their contacts, but no mandatory mask-wearing, and physical distancing adopted after June 21, 2020*. The fraction of successfully followed-up contacts was assumed to be, on average, equal to 50% of all individuals linked to an infected case. Thus, the number of individuals contacted per each case was a function of the size of the contact network of the infected case. Followed-up contacts move to quarantine status; individuals enrolled in the contact tracing procedures stay in quarantine when they become infected. Two scenarios were simulated:
  1. Baseline | 70% of infected people enrolled.
  2. Baseline | 50% of infected people enrolled.
  The fraction of enrolled cases was set to proxy two contact-tracing systems with middle and high performance. Mask-wearing and physical distancing were set to 20% to represent the protective behavior adopted by individuals when these NPIs are not mandatory.
D. *Opening all schools in KSA as of December 1, 2020*. The hypothetical scenario simulates the effect of four school opening strategies:
  1. No mandatory of mask-wearing and physical distancing at national level and in schools.
  2. Mandatory mask-wearing and physical distancing at national level with adherence set to 50%. No mask-wearing and physical distancing policies adopted in schools during activities.
  3. Mandatory mask-wearing and physical distancing at national level with set to 50%. Mask-wearing and physical distancing policies adopted in secondary schools and universities. We assumed no mask-wearing and no physical distancing among children enrolled in nurseries due to the difficulty to enforce the COVID-19 guidance in very young children. Two hypothetical scenarios were tested:
    ▪ Mask-wearing and physical distance set to 50% for secondary schools and universities. (middle adherence level).
    ▪ Mask-wearing and physical distance set to 70% for secondary schools and universities. (high adherence level).
E. *Lifting the international travel ban*. We calculated the fraction of imported cases if the international travel ban would be lifted after January 1, 2021. The number of imported cases was calculated using the number of estimated yearly arrivals [58] and the fraction of infected people among returning travelers [59]. Three hypothetical scenarios were tested:
  1. No quarantine for travelers.
  2. Mandatory quarantine with 50% adherence (middle adherence level).
  3. Mandatory quarantine with 80% adherence (high adherence level).

### Simulation runs and sensitivity analysis

The model ran for 84 weeks (March 2, 2020–July 31, 2021), with NPI scenarios enacted after June 21, 2020, when lockdowns were lifted. The KSA-IBM reproduced the interventions implemented in KSA from March 2 to June 21, 2020, and the same period was also used to calibrate the model and set transmission parameters (Additional file 1). The output of each scenario was based on 500 simulations. The coverage and efficacy of interventions are based on the most updated information about the COVID-19 pandemic. A sensitivity analysis was carried out to identify those NPI parameters that had a high impact on the model’s uncertainty. The sensitivity analysis was performed by varying nine NPI parameters as follows: reduction of interaction among people due to lockdown; adherence to mask-wearing; physical distancing; self-isolation; travel quarantine for travelers; protection against infection provided by masks and physical distancing; contact tracing enrolment and fraction of contact traced (Additional file 1) [45, 51, 55]. The number of cases was the model output used to perform the sensitivity analysis.

## Results

### Lifting lockdowns with no NPIs

**Fig. 1:**
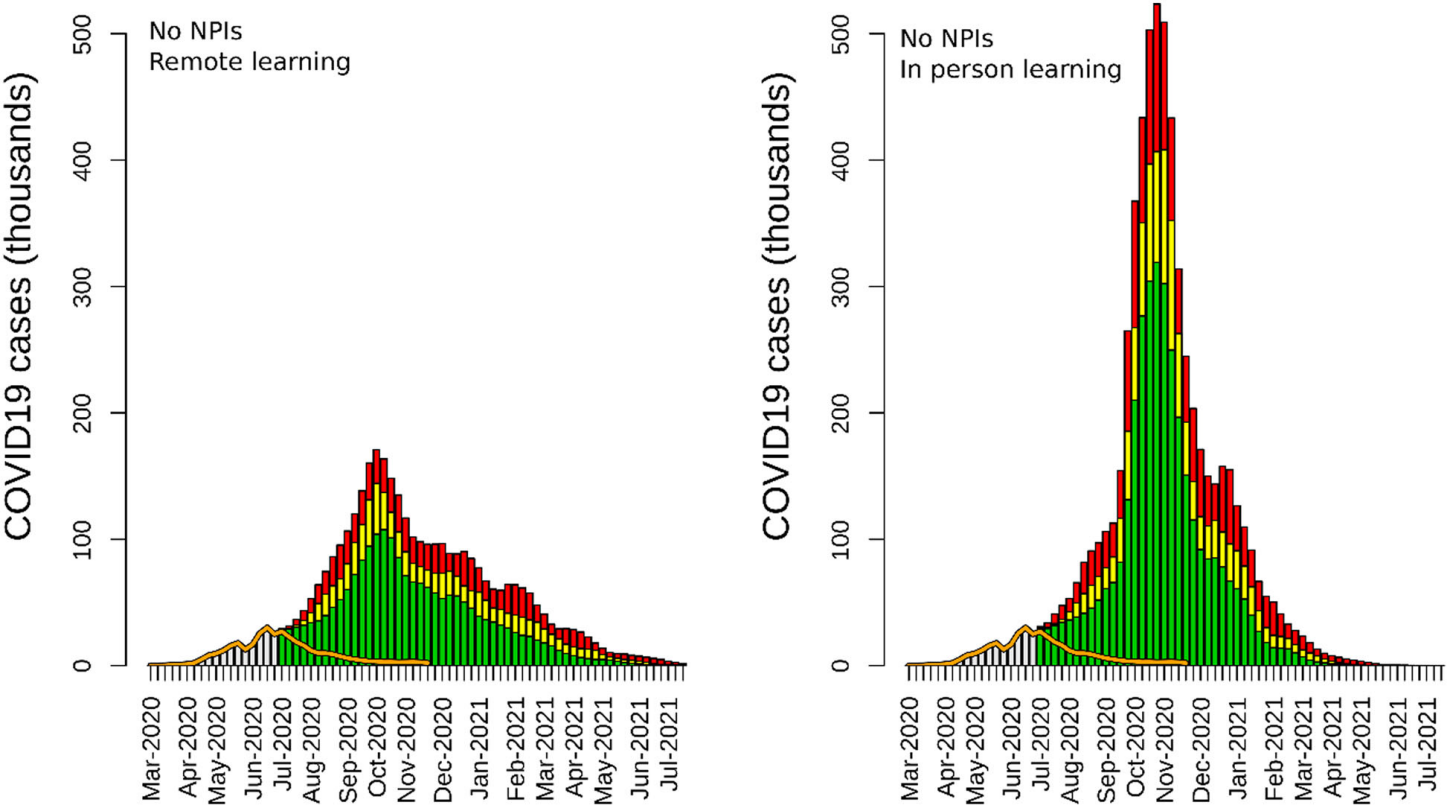
Epidemic curves of the scenarios of remote education vs. in-person education since June 21, 2020, without mandatory NPIs (mask-wearing and physical distancing, and contact tracing). The yellow bars are the mean values of the simulation, and the green and red bars are the 95% CI. The orange line represents the reported number of cases until November 15, 2020. The gray bar represents the number of cases reported until June 21, 2020

The model results showed that lifting lockdown measures at the national level without mandating and enforcing mask-wearing and physical distancing or having a functional contact tracing system in place would have caused a rapid increase in COVID-19 cases, hospitalizations, and deaths. A scenario without NPIs after June 21, 2020, as well as with remote education resulted in a total of 2,832,645 (95% credible interval [95% CI] 2,164,487–3,664,242) cases, 368,244 (95% CI: 281,383–476,351) hospitalizations, and 45,889 (95% CI: 35,065–59,361) deaths (Table 1). In this non-NPIs scenario, the epidemic peaked in November 2020, resulting in a large-scale outbreak in KSA, which resolved by June 2021 (Fig. 1).

**Table 1:** Results of the KSA-IBM for all the simulated scenarios (from June 21, 2020, to June 21, 2021). The table shows the number of reported cases, hospitalized cases, and deaths. The number in the bracket represents the 95% credible interval

Scenario	Reported cases (95% CI)	Hospitalizations (95% CI)	Deaths (95% CI)
*Mask-wearing and physical distancing not enforced*			
Mask 0%| Distancing 0% (Remote education)	2,832,645 (2,164,487–3,664,242)	368,244 (281,383–476,351)	45,889 (35,065–59,361)
Mask 0%| Distancing 0% (In-person education)	4,824,065 (3,673,775–6,335,423)	627,128 (477,591–823,605)	78,150 (59,515–102,634)
*Enforced mask-wearing and physical distancing*			
Mask 50% | Distancing 50%	697,311 (519,984–917,690)	90,650 (67,598–119,300)	11,296 (8424–14,867)
Mask 80% | Distancing 50%	397,361 (347,641–509,685)	51,657 (45,193–66,259)	6437 (5632–8257)
Mask 50% | Distancing 70%	360,308 (330,392–418,019)	46,840 (42,951–54,342)	5837 (5352–6772)
Mask 80% | Distancing 70%	304,858 (298,169–316,210)	39,632 (38,762–41,107)	4939 (4830–5123)
Mask 20% | Distancing 20% Contact tracing 50%	1,354,458 (904,170–2,005,074)	176,080 (117,542–260,660)	21,942 (14,648–32,482)
Mask 20% | Distancing 20%| Contact tracing 70%	616,643 (473,405–864,682)	80,164 (61,543–112,409)	9990 (7669–14,008)
*Resuming in-person education*^a^			
In-person education (No NPIs in schools)^a^	4,801,416 (3,816,009–6,258,459)	624,184 (496,081–813,600)	77,783 (61,819–101,387)
In-person education (Mask 50% | Distancing 50% in schools)	3,539,897 (2,859,676–4,488,397)	460,187 (371,758–583,492)	57,346 (46,327–72,712)
In-person education (Mask 70% | Distancing 70% in schools)	2,304,308 (1,790,133–3,086,625)	299,560 (232,717–401,261)	37,330 (29,100–50,013)
*Lifting international travel ban*^b^			
International travel ban lifted (No quarantine)^a^	3,062,395 (2,758,885–3,476,121)	398,111 (358,655–451,896)	49,611 (44,694–56,313)
International travel ban lifted (Quarantine: 50%)^a^	384,100 (346,977–469,237)	49,933 (45,107–61,001)	6222 (5621–7602)
International travel ban lifted (Quarantine: 80%)^a^	349,409 (327,304–400,363)	45,423 (42,550–52,047)	5660 (5302–6486)

^a^The model scenario has compliance with mandatory mask-wearing, and physical distancing set to 50%, and contact tracing to 50% of reported cases

**Fig. 2:**
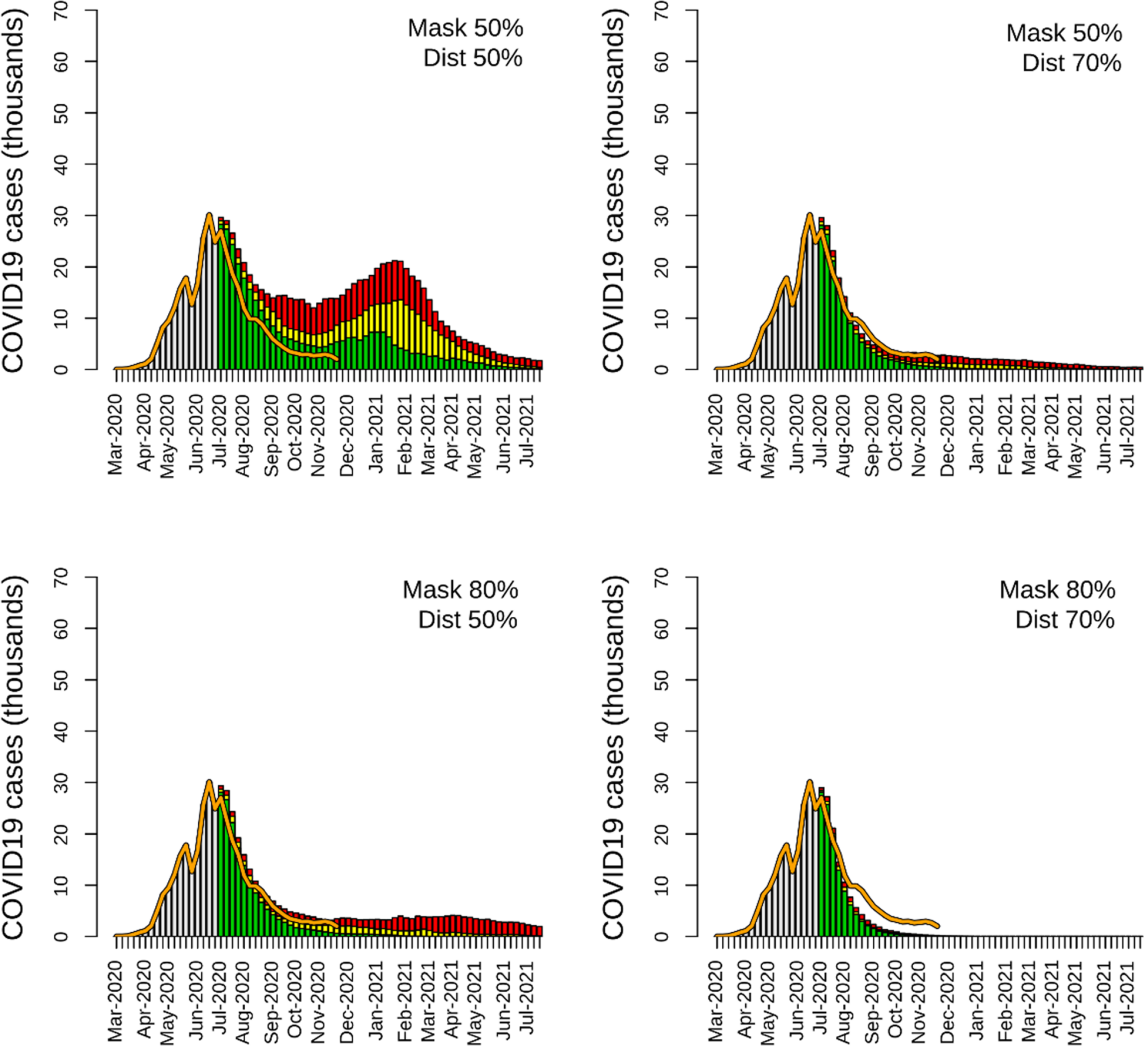
Epidemic curves of the four scenarios with mandatory mask-wearing and physical distancing. The yellow bars are the mean values of the simulation, and the green and red bars are the 95% credible interval. The orange line represents the reported number of cases until November 15, 2020. The gray bar represents the number of cases reported until June 21, 2020

**Fig. 3:**
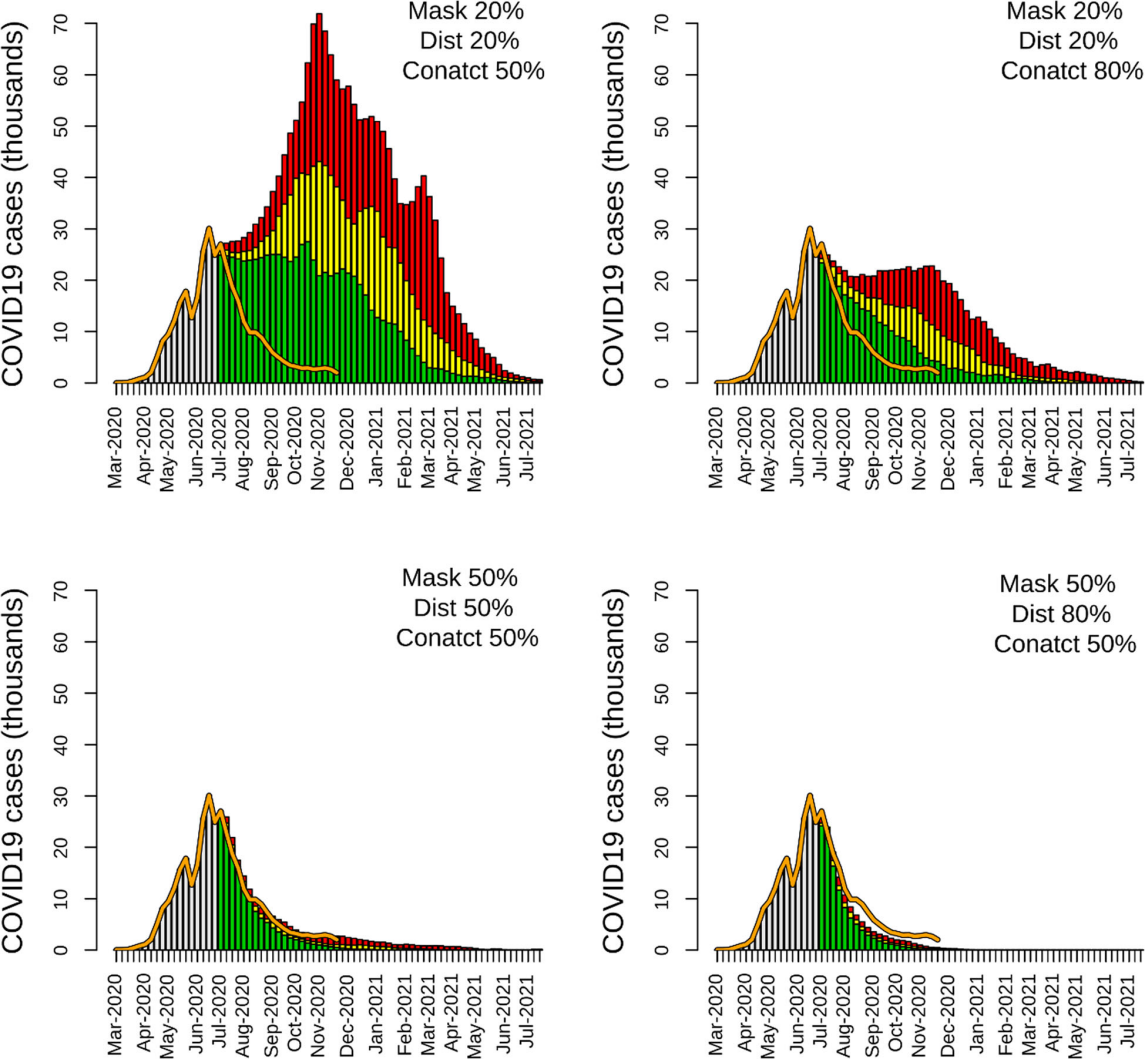
Epidemic curves of the two scenarios simulating contact tracing (from June 21, 2020, to June 21, 2021). The yellow bars are the mean values of the simulation, and the green and red bars are the 95% credible interval. The orange line represents the reported number of cases until November 20, 2020. The gray bar represents the number of cases reported until June 21, 2020

The scenarios in which NPIs were adopted after June 21, 2020, showed high effectiveness of mask-wearing, physical distancing, and contact tracing in reducing the circulation of the virus among the simulated population. A 50% adherence to NPI guidance promoting personal protection (mask-wearing and physical distancing) was able to substantially reduce reported cases, hospitalizations, and deaths caused by COVID-19. In a ‘Mask 50% | Distancing 50%’ scenario, the number of total reported cases was 697,311 (95% CI: 519,984–917,690) throughout the entire simulation period, 75.3% lower than the non-NPI scenario (Table 1). This lower number of reported cases resulted in a reduction of hospitalizations (90,650; 95% CI: 67,598–119,300) and deaths (11,296; 95% CI: 8424–14,867) compared to the non-NPI scenario (Table 1). The epidemic curve of the ‘Mask 50% | Distancing 50%’ scenario was characterized by a rapid decline of reported cases in June 2020, followed by a resurgence during winter of 2020/2021 (Fig. 2). The epidemic lasted for the simulation's entire duration, with a low number of cases reported in July 2021 (Fig. 2). In the scenarios with a high level of personal protection, the number of reported cases drastically declined to a low number of reported cases. The ‘Mask 50% | Distancing 70%’ and ‘Mask 80% | Distancing 70%’ scenarios had a similar epidemic curve, with a long tail of a small number of reported cases until the end of the simulation period (Fig. 2). Both tested NPI scenarios had epidemic curves that were close to the observed KSA epidemic curve (Fig. 2). In the scenario with high level of personal protection, ‘Mask 80% | Distancing 70%’, the epidemic ended in November 2020, with a reduction of reported cases (304,858; 95% CI: 298,169–316,210), hospitalizations (39,632; 95% CI: 38,762–41,107), and deaths (4939; 95% CI: 4830–5123) close to 90% compared to the non-NPI scenario. The number of reported cases, estimated with the ‘Mask 80% | Distancing 70%’ scenario, declined faster than the observed data. The simulations showed that contact tracing needs to reach a high level of coverage to mitigate a COVID-19 epidemic in KSA. Contact tracing with coverage equal to 50%, combined with a low level of personal-protection NPI adherence, was not able to drastically reduce the outcome of the epidemic. A ‘Mask 20% | Distancing 20% | Contact 50%’ scenario was characterized by a short plateau period of a few weeks after the lockdown lifted, followed by a resurgence of cases (Fig. 3). Compared to the non-NPI scenario, contact tracing coverage of 50% with low personal protection behavior was able to halve the number of reported cases (1,354,458; 95% CI: 904,170–2,005,074), hospitalizations (176,080; 95% CI: 117,542–260,660), and deaths (21,942; 955 CI: 14,648–32,482) (Table 1). At a high level of coverage (‘Mask 20% | Distancing 20% | Contact 80%’ scenario), a NPI strategy based only on contact tracing was also able to reduce the size of the epidemic. However, when contact tracing was set at 80%, the case decline was less marked than those estimated in the scenarios with a high level of personal protection (Figs. 2 and 3). The two scenarios in which the three types of NPIs (mask-wearing, physical distancing, and contact tracing) were implemented together showed a high level of transmission reduction even with a low level of contact tracing coverage and personal protection behavior (Fig. 3, Table 1). The estimates obtained with the ‘Mask 50% | Distancing 50% | Contact 50%’ scenarios were similar to KSA’s observed data and the results of the ‘Mask 50% | Distancing 80%’ and ‘Mask 70% | Distancing 50%|’ scenarios (Table 1).

### Resuming in-person education

**Fig. 4:**
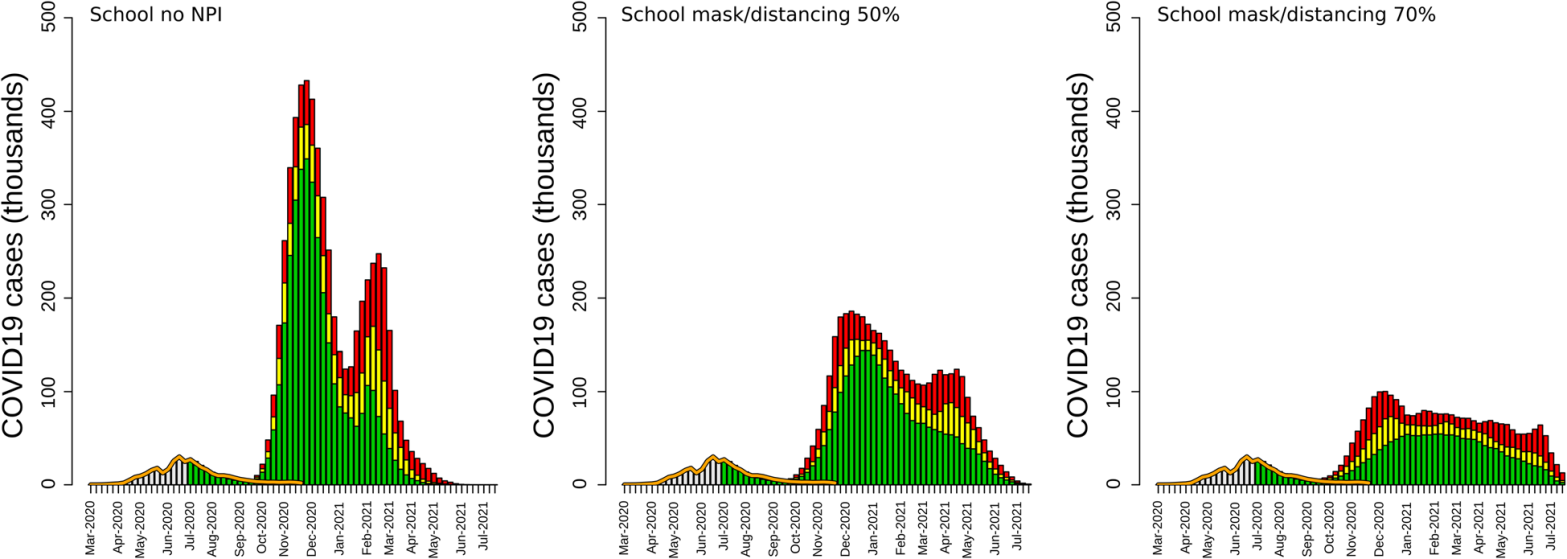
Epidemic curves of scenarios simulating resuming of in-person education with different levels of personal protection based on mask-wearing and physical distancing (from June 21, 2020, to June 21, 2021). The panel shows estimates from a scenario with no personal protection (left), with mask-wearing and physical distancing performed by 50% (center) and 70% (right) of the school population. In the graphs, the yellow bars are the mean values of the simulation, and the green and red bars are the 95% credible interval. The orange line represents the reported number of cases until November 20, 2020. The gray bar represents the number of cases reported until June 21, 2020

Resuming in-person education resulted in a rapid increase in reported cases in all scenarios (Figs. 1 and 4). Performing in-person teaching without mandatory mask-wearing and physical distancing, and without contact tracing resulted in an epidemic curve characterized by 4,824,065 (95% CI: 3,673,775–6,335,423) cases, 627,128 (95% CI: 477,591–823,605) hospitalizations, and 78,150 (95%: 59,515–102,634) deaths by the end of July 2021 (Table 1, Fig. 1). These estimates were approximately 40% higher than those obtained when no personal protection NPIs were enforced but with remote education; in this scenario, the peak was reached in November 2020. When in-person education was allowed with a contact tracing system and mandatory self-protection at the national level but not enforced in schools, the number of estimated reported cases was 4,801,416 (95% CI: 3,816,009–6,258,459). In this scenario, the peak of transmission occurred in December 2020, followed by a smaller peak in February 2021 (Table 1, Fig. 4). Mask-wearing and physical distancing applied to schools were able to mitigate the effect of in-person education on the transmission of the virus. When mask-wearing and physical distancing were performed by 50% of individuals in school, the number of reported cases dropped by 26.2 % (3,539,897; 95% CI: 2,859,676–4,488,397) (Table 1), with the peak of transmission occurring in January 2021 and the end of the epidemic occurring at the end of July 2021. Setting mask-wearing and physical distancing adherence at 70% in schools resulted in a reduction of 52% of cases the scenarios with no interventions in schools (Table 1). The epidemic curve of this scenario showed a ‘flattened’ shaped curve. In this scenario, the number of cases remained constant from January to March 2021, and, eventually, rapidly declined after May 2021. In scenarios with NPIs in schools, hospitalizations and deaths were reduced by 25% and 50%, when NPI adherence was set to 50% and 70%, respectively.

### Lifting the international travel ban

**Fig. 5:**
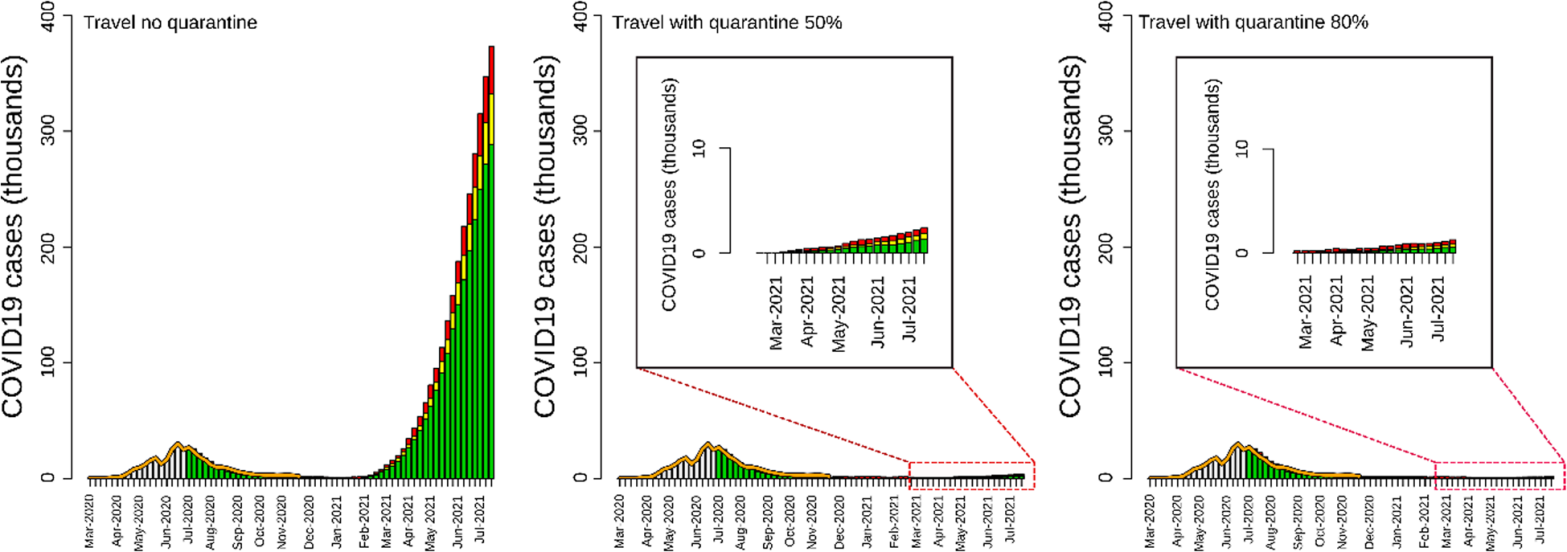
Epidemic curves of scenarios simulating resuming of international travel with different levels of quarantine (from June 21, 2020, to June 21, 2021). The panel shows estimates from a scenario with no quarantine (left), 50% of travelers in quarantine (center), and 70% of travelers in quarantine (right). In the graphs, the yellow bars are the mean values of the simulation, and the green and red bars are the 95% credible interval. The orange line represents the reported number of cases until November 20, 2020. The gray bar represents the number of cases reported until June 21, 2020

The model’s estimates showed that lifting the travel ban without quarantine could drastically increase cases, hospitalizations, and deaths (Table 1, Fig. 5). The scenarios resulted in an estimated number of cases equal to 3,062,395 (2,758,885–3,476,121), 398,111 (358,655–451,896) hospitalizations, and 49,611 (44,694–56,313) deaths (Table 1, Fig. 5). The model showed that mandatory quarantine was able to reduce the number of cases, hospitalizations, and deaths by 87%, when quarantine adherence was set to 50%, and by 88.5% when adherence was set to 80%.

### Sensitivity analysis

The results obtained from the sensitivity analysis showed that the uncertainty of the model was highly affected by a variation in reduced interaction among people due to lockdown, adherence to self-isolation rules, as well as protection provided by masks and physical distancing. Case reduction was strongly linked to lockdown and self-isolation. The level of protection offered by mask-wearing and physical distancing were the main drivers of the impact of these two NPIs on the modeled epidemic curve’s size (Additional file 1: Fig. S1).

## Discussion

Our study showed that lifting KSA’s lockdown without NPIs could have resulted in a large COVID-19 outbreak with important consequences for the country’s health system. Therefore, KSA’s strategy of implementing strict NPIs while lifting the lockdown resulted in a substantial reduction in COVID-19 case numbers. Lifting lockdowns without mandatory self-protection and contact tracing would have caused an estimated 4 million cases by July 2021. The KSA-IBM also highlighted the importance of mandating and enforcing NPIs when exploring the possibility of resuming in-person teaching and lifting the international travel ban.

Mask-wearing, physical distancing, and contact tracing have been among the main NPIs to reduce and control the COVID-19 burden in several countries since the beginning of the pandemic [12]. Mathematical models based on ongoing, in-country COVID-19 response efforts and epidemiological data have highlighted how mask-wearing, physical distancing, and contact tracing can substantially reduce the virus’s circulation [12, 60, 61]. Our model results show that circulation of SARS-CoV-2 was not able to be reduced by just NPIs alone when coverage and adherence of these were not optimal. Our prediction model shows that high coverage mask-wearing without adhering to physical distancing and without effective contact tracing would not avoid a large outbreak after lifting lockdown measures. Prior models have shown that the effectiveness of one NPI, such as mask-wearing or physical distancing, can only be achieved when a very high proportion (i.e., 85–95%) of the population adhered to recommended norms [25]. In reality, due to many reasons (e.g., individual behavior, social-economic status, cultural background), adherence to mask-wearing, physical distancing, and other NPIs is not uniform across the population, reducing NPI coverage and effectiveness [62, 63]. Therefore, multiple NPIs need to be implemented concurrently to protect people from SARS-CoV-2 infection. Our model shows that when multiple NPIs were enforced in the KSA, their synergetic effect was able to reduce virus transmission even when adherence was set at a conservative 50%. These model results agree with the findings of studies focused on assessing the effect of NPIs on SARS-CoV-2 spread [60, 64, 65]. Our model results show great concurrence to KSA’s epidemic curve when mask-wearing and physical distancing were adhered to by half of the population, and contact tracing was able to identify 50% of cases and their contacts. A multi-country analysis based on data from 130 countries has highlighted how the synergic effect of NPIs was able to control the spread of the disease [12].

Children have been shown to have a lower probability of having symptomatic COVID-19 when infected with SARS-CoV-2 [66]. Yet, their viral shedding and infectiousness do not seem different from those of other age groups when infected [67]. Thus, children were identified as a possible key player in the transmission of the virus at the beginning of the pandemic [68]. Their role in the transmission dynamics of other highly infectious diseases such as influenza is well documented [69], and school closings have been shown to drastically decrease the number of influenza cases during an epidemic [70]. Consequently, many countries adopted a school closing approach and opted for remote (or virtual) schooling as an NPI to mitigate SARS-CoV2 spread [12]. While models have shown that remote schooling can drastically reduce the number of COVID-19 cases, school closings can have a negative impact on child learning and their physical and mental health [71, 72], as well as a negative impact on household income (e.g., if a parent or caregiver has to become part-time or quit their job in order to take care and even home-school their child) [73, 74]. To avoid these negative effects of school closings, some countries opted to maintain in-person teaching, but implementing various NPIs (e.g., hybrid in-person and virtual teaching, limiting the number of students in a classroom, physical distancing students’ desks, canceling physical activities) to reduce contact between staff and students, possible exposure to and spread of the virus. Recent studies have shown that schools' role in the spread of SARS-CoV-2 has become less important when mask-wearing and physical distancing interventions are fully and effectively implemented [75, 76]. Our model shows that resuming in-person teaching in the KSA could cause a drastic surge of COVID-19 cases in scenarios in which no population-wide NPIs are enforced. In a scenario where multiple NPIs are enforced in the general population but not in schools, the effect of resuming in-person teaching on such resurgence was reduced, but would likely still result in a large outbreak with 40% more cases compared to a scenario where schooling is fully remote. When physical distancing and mask-wearing were adopted by a large fraction of school students and staff, the surge of cases caused by allowing in-person teaching was minimized, preventing around 50% of cases compared to a scenario where no NPIs are enforced in schools. Thus, lack of mask-wearing among students was one of the reasons causing a large COVID-19 outbreak in a high school in Israel a few days after in-person teaching had been fully re-established [77].

As of March 2021, KSA has partially opened to international flights. However, people traveling to the KSA had to quarantine at their points of entry before being allowed to freely move about the country. Our study shows that lifting the international travel ban could cause a rapid increase in reported cases, hospitalizations, and deaths due to the introduction of infected individuals from other countries—as recently shown in the UAE [78]. The KSA-IBM shows that mandatory quarantine for international travelers drastically reduces the probability of COVID-19 outbreaks, findings which are in agreement with the results obtained by others assessing the importance of quarantine on SARS-CoV-2 spread following international travel [79]. Limiting the possibility of outbreak occurrence due to international travelers is crucial for countries that have successfully reduced and maintained the number of in-country cases close to zero. After dramatically reducing virus circulation in their territories using NPIs, Australia and New Zealand have implemented strict quarantine rules for travelers to avoid further COVID-19 outbreaks [7]. More recently, international travel has been shown to have majorly contributed to the global spread of new SARS-CoV-2 variants such as the delta variant; how these new variants affect the roll-out of COVID-19 vaccines is a concern [80]. The fear of the new SARS-CoV-2 variants spreading even further has forced countries to consider and enact travel bans from countries where the new variants are circulating and prevalent.

Since January 6, 2021, a resurgence of cases has been recorded in the KSA, raising concern of future outbreaks [81]. This resurgence has been attributed to a reduction in population adherence of NPIs, perhaps due to ‘COVID-19 fatigue’ [82]. The KSA government has already asked the population to comply with NPI guidance to avoid more strict enforcement, curfews, and lockdowns [82].

Our study has some limitations that should be noted. First, the social network was not based on field data on human interaction collected in the KSA, but created using common characteristics found studying human interactions in different settings merged with KSA demographic data. The movement of people in the country was simulated using a gravity model based on general movement rules and not from movement data of the KSA population, such as mobile phone data. The use of approximations to represent the interaction of the KSA population may have impacted the precision of our model in forecasting the progression of the epidemic in the country. Second, the transmission of the virus was calibrated using weekly reported cases. Case reporting has not shown to be consistent in many countries, thereby underestimating the size of COVID-19 epidemics. Thus, the precision of the IBM-KSA prediction was linked to the performance of the KSA case reporting system. Third, our model was developed before the SARS-CoV-2 delta variant was identified and prevalent in KSA; the first KSA case due to the delta variant was reported in August 2021, which is outside the time period of our model. The fraction of cases due to the delta variant in KSA is currently unknown.

Our model’s results showed that even with strict NPI enforcement and remote schooling, the circulation of SARS-CoV-2 in KSA has a high probability of continuing beyond July 2021. This is due to the high fraction of the population who are either not vaccinated or still naïve to the virus. Our results highlight that without a high fraction of the population protected by a vaccine and continued high coverage and adherence to NPIs, the circulation of the virus in the KSA could last for a long time, and the country will have to rely on NPIs and high population adherence to NPIs to avoid a resurgence of COVID-19.

## Conclusion

Our study showed how enforcing multiple NPIs has helped the KSA to reduce and control the burden of COVID-19 on its population. Resuming in-person teaching and lifting the international travel ban should be considered only if the country can enforce high coverage and adherence to mask-wearing and physical distancing in schools, as well as force strict quarantine of international travelers at points of entry. In the absence of high vaccine coverage, the KSA government will have to continue to enforce multiple NPIs to avoid a resurgence of COVID-19.

## Supplementary Information

**Additional file 1. **Description of model structure and sensitivity analysis results. **Table S1**. KSA-IBM network parameters. **Table S2**. COVID-19 transmission parameters of the KSA-IBM. **Table S3**. Articles used to compute the probability transmission range used to calibrate IBM-KSA’s transmission parameter. **Figure S1.** Results of the sensitivity analysis. **Figure S2.** Effective reproductive (*Rt*) number of IBM-KSA scenarios.

## Data Availability

Data and model code are available from the corresponding author on reasonable request.

## Abbreviations

KSA: Kingdom of Saudi Arabia
NPI: Non-pharmaceutical intervention
IBM-KSA: Individual-based model representing the country population
MENA: Middle East and North Africa countries
CFR: Case fatality rate
