# Supplementary material for: Estimating the effect of non-pharmaceutical interventions to mitigate COVID-19 spread in Saudi Arabia
Source: BMC Med. 2022 Feb 7;20:51. doi: 10.1186/s12916-022-02232-4 (PMC8818364; doi:10.1186/s12916-022-02232-4)
Supplement: Supplementary file 1 — Additional file 1. Description of model structure and sensitivity analysis results. Table S1. KSA-IBM network parameters. Table S2. COVID-19 transmission parameters of the KSA-IBM. Table S3. Articles used to compute the probability transmission range used to calibrate IBM-KSA’s transmission parameter. Figure S1. Results of the sensitivity analysis. Figure S2. Effective reproductive (Rt) number of IBM-KSA scenarios. [file 12916_2022_2232_MOESM1_ESM.docx]

**DATA DICTIONARY**

*Demographic data*

Data on the Kingdom of Saudi Arabia (KSA) population at high spatial resolution was obtained from WorldPop (<https://www.worldpop.org/project/categories?id=18>). WorldPop provides population estimates broken down by gender and age groupings (including 0–1 years and by 5-year ranges up to 80+ years) at a resolution of 100 meters. The data obtained from WorldPop was used to estimate the size of the spatial-explicit human network. Average house size and house age composition were obtained from the KSA General Authority of Statistic’s website (<https://www.stats.gov.sa/en/node>)

*Education data*

KSA education data was obtained from difference sources. The average class size number was gathered from the Organization for Economic Co-operation and Development (OECD) [30]. Data on school enrolment for primary school, secondary school, and university was downloaded from World Bank Open Data [31]. Pre-school information was gathered from Rabaah et al. 2016 [32].

*Human Settlements*

To identify human settlements at high resolution, the population density map created by Facebook (<https://dataforgood.fb.com/tools/population-density-maps/>) was used. This map identifies areas with buildings at a resolution of 30 meters, as updated to 2019. Population density map data were analyzed using spatial analysis techniques to identify the position of human settlements. KSA was divided in 10x10Km squares and only those in which human settlements were present were included in the model. The total number of squares used in the model was equal to 2,057. We calculated the number of people living in each square using WorldPop data. The 2,057 squares represent the settlements of the model.

*Travel Time Between Settlements*

The movement of people between squares was based on population size and travel times, as explained below. Population sizes were calculated using WorldPop and Facebook data, as described previously. Travel times were calculated using the friction surface map created by the Malaria Atlas Project (MAP) [33]. The friction surface map contains the time that a person spends passing through a map pixel based on estimated land-based travel speeds at a resolution of 1 km. Using the friction surface map estimation, we calculated the travel time between settlements.

*Changes of movement pattern*

Data on movement pattern variations as a results of non-pharmaceutical interventions (NPIs) being implemented during the pandemic was obtained from Community Mobility Reports made by Google [34].

**KSA-IBM MODEL STRUCTURE**

The following section provides a detailed description of the KSA-IBM human social network components. A summary of the KSA-IBM’s parameters and data sources is provided in Tables S1 and Table S2. Given the transmission dynamics of COVID-19 and the time lag of the surveillance to report cases, the model ran on a weekly time step. Weekly estimates made it easier to compare KSA-IBM estimates with real data.

*Representing the Human Social Network (Intra-settlement Network).*

The KSA-IBM model captured the high heterogeneity of the contact network among people, which is the key driver of disease spread in communities. The KSA-IBM network was built using the “scale-free” and “small-world” characteristics described for several social networks. A scale-free network has a high fraction of nodes connected to a low number of other nodes, and a few nodes have a high number of links. The nodes (individuals) that have high connectivity are often called “super-spreaders.” The link distribution among nodes in the scale-free network followed a power-law distribution:

$$p\left( x \right)=x^{-\alpha}$$

where x is the number of links of a node, p(x) is the cumulative distribution, and the exponent α is the scaling factor. For human social networks, the power-law distributions have exponent α values ranging from 2 to 3 [35]. A network has a “small-world” characteristic when two nodes in the network can reach each other through a short sequence of connected nodes (called a “short path”) [53].

The infection probability of a naïve individual by an infectious one was linked to the duration of their interaction. To capture transmissible contact duration a weighted network was used [36]. In a weighted network, all links have a weight that describes the strength of the transmissible contact between two nodes. In the KSA-IBM, the weight of a link was represented by the duration of transmissible contact in minutes. The distribution of interaction duration among people was quite heterogeneous and was characterized by many short-life interactions (< 10 minutes) and few interactions lasting for a long time (> 1 hour). The distribution of interaction duration followed a power-law distribution where the coefficient (α ~1.5) was calculated using real data collected in different settings [36–38]. This coefficient is generally consistent across countries and settings.

Interactions among individuals occurred in specific locations, which may have a key role in the spread of disease agents. The locations in which people spend most of their daily time are households, workplaces, and schools [39, 40]. However, interactions outside the routine locations (e.g., markets, restaurants, and cinemas) are at the base of the small-world characteristic of human social networks. Age is another important factor that shapes the social network of an individual. People tend to have more interaction with individuals of the same age that they meet at school, workplaces, or in recreational locations [41]. Thus, to be sure that the KSA-IBM accurately described the interaction among people, the KSA-IBM included the distribution of interaction among individuals, interaction duration and location, and the effect of the individuals’ ages.

*Creation of contact network*

After the population size and distribution of age groups of a settlement were estimated, a contact network was created using the following steps:

1. Households were created using the mean size of the family cluster. The number of people living together in each house was determined using a Poisson distribution, with the mean equal to the mean size of household inhabitants of KSA (i.e., 6.4 members). Contact networks of each individual started with links to household members.
2. Links outside the household were calculated for each individual based on a power-law distribution with α = 2.5. The duration of each contact was estimated using a power-law distribution with α = 1.5.
3. The formulation of a power-law distribution requires indicating a minimum value for *x*. The minimum number of contacts for each individual was equal to the number of household members. The minimum duration of a contact was equal to 1 minute. The maximum number of contacts was naturally constrained by the duration of contacts, where the total cumulative time of direct contact could not exceed 24 hours per day on a week time window (week is the KSA-IBM time step).
4. Each individual was linked to other individuals following contact matrices reported in Prem et al.[41].
5. For all individuals attending schools, a number of links extracted from a Poisson distribution with mean equal to the average class size were created. The links were connected with individuals of same age and from same settlement (square).
6. A location attribute was assigned to all links. We assigned four types: household, workplace, school, or other. The probability of assigning a link to a particular group was based on demographic characteristics of the KSA population.

*Representing Human Movement Among Settlements*

The extra-settlement spread of SARS-CoV-2 in the KSA-IBM was captured using a weighted network that links settlements. The weight of each link was determined by the estimated flux of people between settlements. We calculated the weight using a gravity model accounting for distance between settlements, travel time, and population size [42, 43]. A gravity model is a modified law of gravitation that, in its simpler formulation (frictionless gravity model), considers the population size of two places and their distance apart to estimate the flow of people between them [44]. This approach is not country-specific but has been used in the past in West Africa. The assumption of the gravity model is that larger settlements attract more people and settlements closer together share more people than distant ones. However, in settings where connections among places are not easy, the model can be adjusted by adding travel times (friction-based gravity model) [44].

The distance and travel time between two settlements was calculated using the population Facebook density map and the surface friction map [33]. The weight of each link was used to calculate the probability that COVID-19 cases will move between two settlements causing the occurrence of a new outbreak. The extra-settlement network was created following these steps:

1. Created a distance matrix among settlements with columns and rows equal to the number of settlements. Each matrix cell contained the distance between two settlements.

2. Created a travel time matrix among settlements with columns and rows equal to the number of settlements. Each matrix cell contained the travel time between two settlements.

3. Built a gravity model merging population data, the distance matrix, and the travel time matrix following the methods described in Kraemer et al. (2019) [43] and Balcan et al. (2009) [43].

4. Recorded the results of the gravity model in a flux matrix among settlements with columns and rows equal to the number of settlements. Each matrix cell contained the estimated flow between two settlements.

Table S1. KSA-IBM network parameters

| Network parameter | Input in the model | Source |
| --- | --- | --- |
| Social network | Estimated using a power-law distribution with α = 2.5 | [35] |
| Interaction duration | Estimated using a power-law distribution with α = 1.5 | [36–38] |
| Age group contact matrix | Estimated from Prem et al. | [41] |
| Extra settlement network |  |  |
| Number of settlements (squares) in KSA | Estimated from the Facebook density map | <https://dataforgood.fb.com/tools/population-density-maps/> |
| Distance among settlements (squares) | Estimated from the Facebook density map | <https://dataforgood.fb.com/tools/population-density-maps/> |
| Travel time among settlements (squares) | Estimated using MAP friction map | [33] |
| Extra settlement people flow | Estimated using the gravity model | [42,43] |

**COVID-19 EPIDEMIC**

In the KSA-IBM, the infectious status of individuals followed the transitions seen in SEIR compartmental models: Susceptible (S) → Exposed (E) → Infectious (I) → Recovered (R). Infectious individuals were also at risk of being hospitalized and dying. The transition from one status to another was a function of pathogen characteristics (e.g., virulence, incubation periods, infectious period, and fatality rate, Table S2) and interaction among individuals (only for S to E). The model account for NPIs and not vaccination campaign, as described in the manuscript’s main narrative.

The model transmission parameter was calculated by calibrating the KSA-IBM on reported cases from March 2, 2020, to June 21, 2020. A Markov chain Monte Carlo (MCMC) method based on 10,000 simulations was applied to identify the best transmission parameter able to reproduce the epidemic curve in KSA. The sensitivity analysis simulations were performed using 8.5 million individuals (25% of the IBM-KSA) to increase computational speed. Each simulation had its transmission probability sampled from a uniform distribution with a 0.0001-0.003 range. The transmission probability range was estimated from published sources describing SARS-CoV-2 transmission chains (Table S3). The best value for the transmission parameter was chosen using the normalized root-mean-square error (NRMSE) [45]. The transmission probability with the lowest NRMSE was used as the transmission parameter in the IBM-KSA.

The model accounts for NPIs and not vaccination campaigns, as described in the manuscript’s main narrative. Effect of movement restriction during the calibration time window was included by reducing the number of non-household links based on Google’s Community Mobility Report [34]. The IBM-KSA run for 500 times for each scenarios.

**Table S2. COVID-19 transmission parameters of the KSA-IBM**

| Parameters | Value | Source |
| --- | --- | --- |
| Transmission per minute | 0.0016 | Calibration |
| Incubation period | 5.8 days (log normal distribution) | [46] |
| Infectious period | 6.5-9.5 (uniform distribution) | [46] |
| Probability to be symptomatic | 0.20 | [47] |
| Fraction of reported symptomatic cases | 80% (mean) | [48] |
| Hospitalization probability | 0.13 | [23,49] |
| Case fatality rate | 0.017 | [23] |

**Sensitivity analysis**

Sensitivity analysis was performed to investigate the effect of parameters used to describe NPIs on model outputs, including model uncertainty, estimated epidemic size, and parameter importance. The Sobol’s index, a variance based method, was computed to identify those parameters with high impact on model uncertainty—the greater the index’s value, the higher the impact on uncertainty. The index was computed using a method based on multivariate regression as described by Lu et al. [50]. Nine NPIs parameters were analyzed in the sensitivity analysis: reduced interaction among people due to lockdown; adherence to mask-wearing; physical distancing; self-isolation; travel quarantine for travellers; protection against infection provided by masks and physical distancing; contact tracing enrolment and fraction of contact traced. A Latin Hypercube Square sampling method was used to create a set of 10,000 randomly generated inputs to use in the sensitivity analysis as described in McKay et al. [51]. For this sampling method, each parameter value was sampled from a uniform distribution of (0, 1) range. A generalized linear model (GLM) with Guassian link was chosen as multivariate regression method. The GLM’s dependent variable was the estimated number of cases obtained by the IBM-KSA run using the set of randomly generated parameters. The independent variables were the values of the parameters use to run the 10,000 simulation.

**Figure S1. Results of the sensitivity analysis.** The figures shows Sobol’s indices and effect on estimated number of cases with (A and C) and without (B and D) accounting for lockdown effect on people interaction.


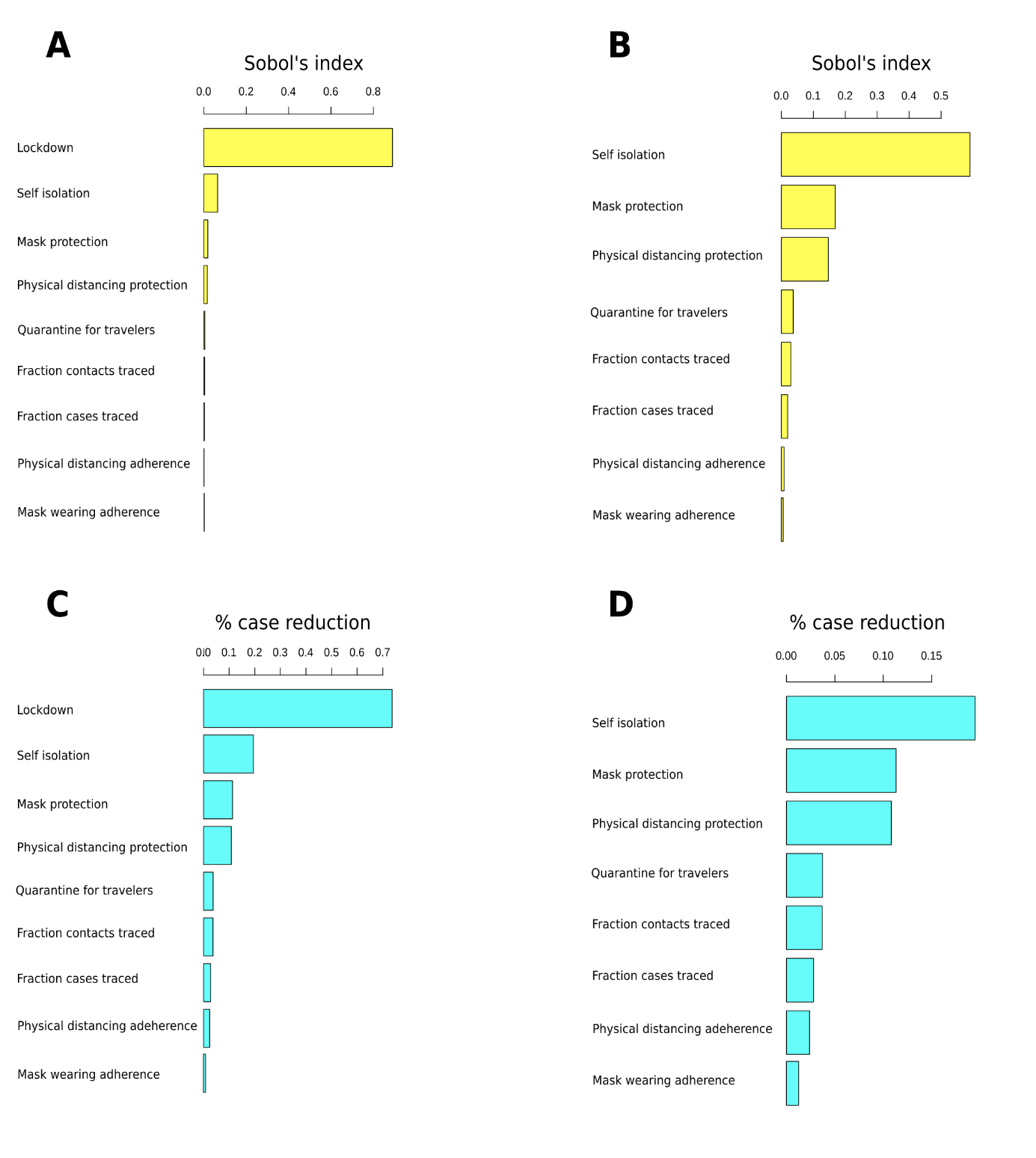


**Figure S2. Effective reproductive (*Rt*) number of IBM-KSA scenarios.** The figure shows in orange the *Rt* of reported cases and in blue estimated *Rt* obtained from IBM-KSA simulations.

**
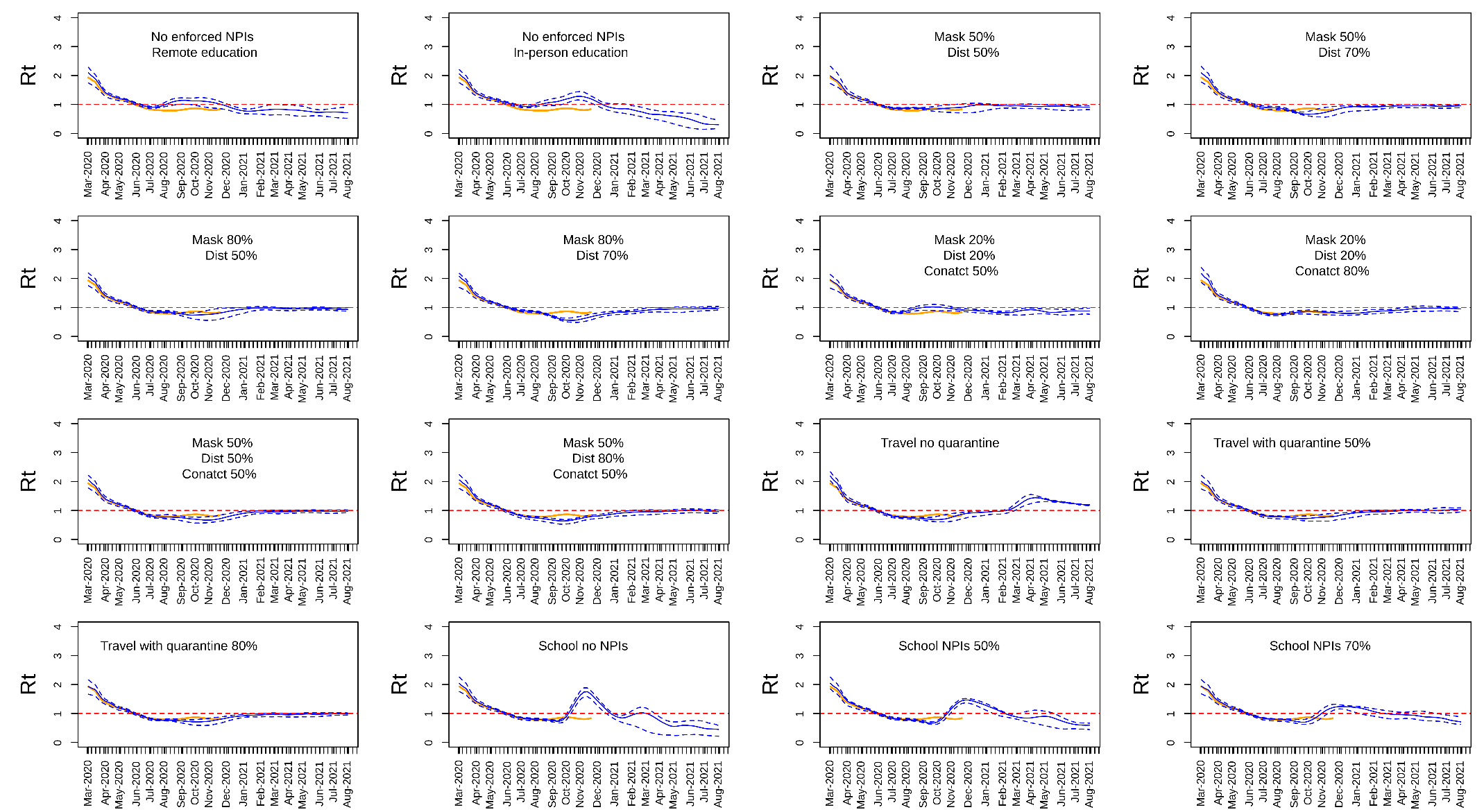
**

**Table S3. Articles used to compute the probability transmission range used to calibrate IBM-KSA’s transmission parameter.**

| **Article reference** | **Transmission setting** | **Link** |
| --- | --- | --- |
| An P, Zhang M. Novel coronavirus SARS-CoV-2: familial spread resulting in COVID-19 pneumonia in a pediatric patient. Diagnostic and Interventional Radiology 2020;26:262. | Household | <https://www.dirjournal.org/en/novel-coronavirus-sars-cov-2-familial-spread-resulting-in-covid-19-pneumonia-in-a-pediatric-patient-132204> |
| Bi Q, Wu Y, Mei S, Ye C, Zou X, Zhang Z, Liu X, Wei L, Truelove SA, Zhang T, Gao W. Epidemiology and transmission of COVID-19 in 391 cases and 1286 of their close contacts in Shenzhen, China: a retrospective cohort study. Lancet Infect Dis. 2020;20:911-9. | Gathering and households | <https://www.thelancet.com/pdfs/journals/laninf/PIIS1473-3099(20)30287-5.pdf> |
| Chan JF, Yuan S, Kok KH, To KK, Chu H, Yang J, Xing F, Liu J, Yip CC, Poon RW, Tsoi HW. A familial cluster of pneumonia associated with the 2019 novel coronavirus indicating person-to-person transmission: a study of a family cluster. Lancet 2020 395:514-23. | Household | <https://www.thelancet.com/journals/lancet/article/PIIS0140-6736(20)30154-9/fulltext> |
| Cheng HY, Jian SW, Liu DP, Ng TC, Huang WT, Lin HH. Contact tracing assessment of COVID-19 transmission dynamics in Taiwan and risk at different exposure periods before and after symptom onset. JAMA Internal Medicine 2020 180:1156-63. | Multiple location | <https://jamanetwork.com/journals/jamainternalmedicine/fullarticle/2765641> |
| Ghinai I, Woods S, Ritger KA, McPherson TD, Black SR, Sparrow L, Fricchione MJ, Kerins JL, Pacilli M, Ruestow PS, Arwady MA. Community transmission of SARS-CoV-2 at two family gatherings—Chicago, Illinois, February–March 2020. Morbidity and Mortality Weekly Report. 2020; 69):446. | Family gathering | <https://www.cdc.gov/mmwr/volumes/69/wr/mm6915e1.htm?s_cid=mm6915e1_w> |
| Giang HT, Shah J, Hung TH, Reda A, Truong LN, Huy NT. The first Vietnamese case of COVID-19 acquired from China. Lancet Infect Dis 2020;20:408-9. | Household | <https://www.thelancet.com/journals/laninf/article/PIIS1473-3099(20)30111-0/fulltex> |
| Hamner L. High SARS-CoV-2 attack rate following exposure at a choir practice—Skagit County, Washington, March 2020. MMWR Morbidity and Mortality Weekly Report. 2020;69. | Choir practice | <https://www.cdc.gov/mmwr/volumes/69/wr/mm6920e2.htm?s_cid=mm6920e2_w> |
| Heinzerling A, Stuckey MJ, Scheuer T, Xu K, Perkins KM, Resseger H, Magill S, Verani JR, Jain S, Acosta M, Epson E. Transmission of COVID-19 to health care personnel during exposures to a hospitalized patient—Solano County, California, February 2020. MMWR Morbidity and Mortality Weekly Report. 2020;69:472. | Hospital | <https://www.cdc.gov/mmwr/volumes/69/wr/mm6915e5.htm?s_cid=mm6915e5_w> |
| Hijnen D, Marzano AV, Eyerich K, GeurtsvanKessel C, Giménez-Arnau AM, Joly P, Vestergaard C, Sticherling M, Schmidt E. SARS-CoV-2 transmission from presymptomatic meeting attendee, Germany. Emerg Infect Dis. 2020;26:1935. | Board meeting | <https://wwwnc.cdc.gov/eid/article/26/8/20-1235_article> |
| Huang R, Xia J, Chen Y, Shan C, Wu C. A family cluster of SARS-CoV-2 infection involving 11 patients in Nanjing, China. Lancet Infect Dis. 2020;20:534-5. | Household | <https://www.thelancet.com/journals/laninf/article/PIIS1473-3099(20)30147-X/fulltext> |
| James A, Eagle L, Phillips C, Hedges DS, Bodenhamer C, Brown R, Wheeler JG, Kirking H. High COVID-19 attack rate among attendees at events at a church—Arkansas, March 2020. MMWR Morb Mortal Wkly Rep. 2020;69:632-635. | Church/Household | <https://www.cdc.gov/mmwr/volumes/69/wr/mm6920e2.htm?s_cid=mm6920e2_w> |
| Jang S, Han SH, Rhee JY. Cluster of coronavirus disease associated with fitness dance classes, South Korea. Emerg Infect Dis. 2020;26:1917. | Dance class | <https://wwwnc.cdc.gov/eid/article/26/8/20-0633_article> |
| Jeong EK, Park O, Park YJ, Park SY, Kim YM, Kim J, Jo J, Kim J, Kim T, Gwack J, Lee J. Coronavirus disease-19: Summary of 2,370 contact investigations of the first 30 cases in the Republic of Korea. Osong Public Health Res Perspect. 2020; 11:81-84. | Gathering and households | <https://www.ncbi.nlm.nih.gov/pmc/articles/PMC7104686/pdf/ophrp-11-81.pdf> |
| Ji LN, Chao S, Wang YJ, Li XJ, Mu XD, Lin MG, Jiang RM. Clinical features of pediatric patients with COVID-19: a report of two family cluster cases. World J Pediatr. 2020;16:267-70. | Household | <https://link.springer.com/article/10.1007/s12519-020-00356-2> |
| Jiehao C, Jin X, Daojiong L, Zhi Y, Lei X, Zhenghai Q, Yuehua Z, Hua Z, Ran J, Pengcheng L, Xiangshi W. A case series of children with 2019 novel coronavirus infection: clinical and epidemiological features. Clin Infect Dis 2020;71:1547-51. | Multiple settings | <https://www.ncbi.nlm.nih.gov/pmc/articles/PMC7108143/> |
| Lim J, Jeon S, Shin HY, Kim MJ, Seong YM, Lee WJ, Choe KW, Kang YM, Lee B, Park SJ. Case of the index patient who caused tertiary transmission of coronavirus disease 2019 in Korea: the application of lopinavir/ritonavir for the treatment of COVID-19 pneumonia monitored by quantitative RT-PCR. J Korean Med Sci 2020;35:e79 | Household, meal | <https://www.ncbi.nlm.nih.gov/pmc/articles/PMC7025910/> |
| Liu T, Gong D, Xiao J, Hu J, He G, Rong Z, Ma W. Cluster infections play important roles in the rapid evolution of COVID-19 transmission: a systematic review. Int J Infect Dis. 2020;99:374-80. | Household | <https://www.ncbi.nlm.nih.gov/pubmed/32194239> |
| Liu Y, Eggo RM, Kucharski AJ. Secondary attack rate and superspreading events for SARS-CoV-2. Lancet. 2020;395:e47. | Household, meal | <https://www.thelancet.com/journals/lancet/article/PIIS0140-6736(20)30462-1/fulltext> |
| Liu YC, Liao CH, Chang CF, Chou CC, Lin YR. A locally transmitted case of SARS-CoV-2 infection in Taiwan. New Engl J Med. 2020;382:1070-2. | Household | <https://www.ncbi.nlm.nih.gov/pmc/articles/PMC7121202/> |
| Liu YF, Li JM, Zhou PH, Liu J, Dong XC, Lyu J, Zhang Y. Analysis on cluster cases of COVID-19 in Tianjin. Zhonghua Liu Xing Bing Xue Za Zhi. 2020;41:653-656. | Household | <https://www.ncbi.nlm.nih.gov/pubmed/32213269> |
| Lu J, Gu J, Li K, Xu C, Su W, Lai Z, Zhou D, Yu C, Xu B, Yang Z. COVID-19 outbreak associated with air conditioning in restaurant, Guangzhou, China, 2020. Emerg Infect Dis. 2020;26:1628-31. | Restaurant | <https://wwwnc.cdc.gov/eid/article/26/7/20-0764_article> |
| Luo Y, Trevathan E, Qian Z, Li Y, Li J, Xiao W, Tu N, Zeng Z, Mo P, Xiong Y, Ye G. Asymptomatic SARS-CoV-2 infection in household contacts of a healthcare provider, Wuhan, China. Emerg Infect Dis. 2020;26:1930-3. | Household | <https://wwwnc.cdc.gov/eid/article/26/8/20-1016_article> |
| McMichael TM, Currie DW, Clark S, Pogosjans S, Kay M, Schwartz NG, Lewis J, Baer A, Kawakami V, Lukoff MD, Ferro J. Epidemiology of Covid-19 in a long-term care facility in King County, Washington. New Engl J Med. 2020;382:2005-11. | Nursy home | <https://www.nejm.org/doi/full/10.1056/NEJMoa2005412?url_ver=Z39.88-2003&rfr_id=ori%3Arid%3Acrossref.org&rfr_dat=cr_pub++0pubmed> |
| Moriarty LF, Plucinski MM, Marston BJ, Kurbatova EV, Knust B, Murray EL, Pesik N, Rose D, Fitter D, Kobayashi M, Toda M. Public health responses to COVID-19 outbreaks on cruise ships—worldwide, February–March 2020. MMWR Morbidity Mortality Weekly Report. 2020;69:347. | Cruise | <https://www.cdc.gov/mmwr/volumes/69/wr/mm6912e3.htm?s_cid=mm6912e3_w> |
| Pan X, Chen D, Xia Y, Wu X, Li T, Ou X, Zhou L, Liu J. Asymptomatic cases in a family cluster with SARS-CoV-2 infection. Lancet Infect Dis. 2020;20:410-1. | Household | <https://www.thelancet.com/pdfs/journals/laninf/PIIS1473-3099(20)30114-6.pdf> |
| Park JY, Han MS, Park KU, Kim JY, Choi EH. First pediatric case of coronavirus disease 2019 in Korea. J Korean Med Sci. 2020;35:e124. | Household | <https://www.ncbi.nlm.nih.gov/pmc/articles/PMC7086086/> |
| Park SY, Kim YM, Yi S, Lee S, Na BJ, Kim CB, Kim JI, Kim HS, Kim YB, Park Y, Huh IS. Coronavirus disease outbreak in call center, South Korea. Emerg Infect Dis. 2020;26:1666-70. | Call center | <https://wwwnc.cdc.gov/eid/article/26/8/20-1274_article> |
| Pung R, Chiew CJ, Young BE, Chin S, Chen MI, Clapham HE, Cook AR, Maurer-Stroh S, Toh MP, Poh C, Low M. Investigation of three clusters of COVID-19 in Singapore: implications for surveillance and response measures. Lancet. 2020;395:1039-46. | Shop, conference, church, and household | <https://www.thelancet.com/journals/lancet/article/PIIS0140-6736(20)30528-6/fulltext> |
| Qiu YY, Wang SQ, Wang XL, Lu WX, Qiao D, Li JB, Gu YY, Zeng Y, Chen Y, Bai WZ, Xu BL. Epidemiological analysis on a family cluster of COVID-19. Zhonghua Liu Xing Bing Xue Za Zhi. 2020;41:494-7. | Household | <https://www.ncbi.nlm.nih.gov/pubmed/32133831> |
| Shim E, Tariq A, Choi W, Lee Y, Chowell G. Transmission potential and severity of COVID-19 in South Korea. Int J Infect Dis. 2020;93:339-44. | Travel | <https://www.sciencedirect.com/science/article/pii/S1201971220301508> |
| Thanh HN, Van TN, Thu HN, Van BN, Thanh BD, Thu HP, Kieu AN, Viet NN, Marks GB, Fox GJ, Nguyen TA. Outbreak investigation for COVID-19 in northern Vietnam. Lancet Infect Dis. 2020;20:535-6. | Multiple settings | <https://www.thelancet.com/journals/laninf/article/PIIS1473-3099(20)30159-6/fulltext> |
| Tong ZD, Tang A, Li KF, Li P, Wang HL, Yi JP, Zhang YL, Yan JB. Potential presymptomatic transmission of SARS-CoV-2, Zhejiang province, China, 2020. Emerg Infect Dis. 2020;26:1052-4. | Household | [https://wwwnc.cdc.gov/eid/article/26/5/20-0198_article","Dong Tong et al](https://wwwnc.cdc.gov/eid/article/26/5/20-0198_article%22,%22Dong%20Tong%20et%20al) |
| Yusef D, Hayajneh W, Awad S, Momany S, Khassawneh B, Samrah S, Obeidat B, Raffee L, Al-Faouri I, Issa AB, Al Zamel H. Large outbreak of coronavirus disease among wedding attendees, Jordan. Emerg Infect Dis. 2020;26:2165-67. | Wedding | <https://wwwnc.cdc.gov/eid/article/26/9/20-1469_article> |
| Zhang J, Tian S, Lou J, Chen Y. Familial cluster of COVID-19 infection from an asymptomatic. Crit Care 2020;24:119. | Household | <https://ccforum.biomedcentral.com/articles/10.1186/s13054-020-2817-7> |
